# UV Radiation and Its Relation to DNA Methylation in Epidermal Cells: A Review

**DOI:** 10.3390/epigenomes4040023

**Published:** 2020-09-30

**Authors:** Naila Francis Paulo de Oliveira, Beatriz Fernandes de Souza, Marina de Castro Coêlho

**Affiliations:** 1 Departamento de Biologia Molecular, Centro de Ciências Exatas e da Natureza, Universidade Federal da Paraíba—UFPB, João Pessoa 58051-900, Brazil; fernandesbioufpb@gmail.com; 2 Programa de Pós Graduação em Odontologia, Centro de Ciências da Saúde, Universidade Federal da Paraíba—UFPB, João Pessoa 58051-900, Brazil; marina.coelho@academico.ufpb.br

**Keywords:** DNA methylation, epigenetic, UV radiation, solar radiation, epidermis, epidermal, epimutation, skin, phototherapy, 5-mC

## Abstract

DNA methylation is the most studied epigenetic mark, and it can be altered by environmental factors. Among these factors, ultraviolet radiation (UV) is little explored within this context. While the relationship between UV radiation and DNA mutations is clear, little is known about the relationship between UV radiation and epimutations. The present study aimed to perform a literature review to determine the influence of artificial or natural (solar) UV radiation on the global and site-specific methylation profile of epidermal cells. A systematic review of the literature was carried out using the databases PubMed, Scopus, Cochrane, and Web of Science. Observational and intervention studies in cultured cells and animal or human models were included. Most studies showed a relationship between UV radiation and changes in the methylation profile, both global and site-specific. Hypermethylation and hypomethylation changes were detected, which varied according to the studied CpG site. In conclusion, UV radiation can alter the DNA methylation profile in epidermal cells derived from the skin. These data can be used as potential biomarkers for environmental exposure and skin diseases, in addition to being targets for treatments. On the other hand, UV radiation (phototherapy) can also be used as a tool to treat skin diseases. Thus, the data suggest that epigenetic homeostasis can be disrupted or restored by exposure to UV radiation according to the applied wavelength.

## 1. Introduction

### 1.1. DNA Methylation

DNA methylation is an epigenetic mark involved in inhibiting gene transcription, inactivating the X chromosome, silencing repetitive elements of the genome, and genomic imprinting. This epigenetic mark is essential for embryonic development and modulation of gene expression throughout life. By definition, epigenetic marks are chemical changes in DNA and histones that are inheritable, reversible, and do not alter the DNA sequence [1].

DNA methylation in eukaryotes occurs mainly at CpG dinucleotides (cytosines that precede guanines and joined by a phosphodiester bond). These dinucleotides are more frequent in gene promoter regions and act as physical barriers for transcription factor binding or as binding sites for methyl binding proteins, thus preventing or decreasing gene transcription. Methylated cytosines (5-methylcytosine, 5-mC) are so common in the eukaryotic genome that they are referred to as the fifth base of DNA [1]. Another base, adenine, has also been found to bear methylation marks (N6-methyladenine, 6-mA) [2] and it has been associated with silencing of repetitive elements (LINE), tumor development, and differentiation of adult and embryonic stem cells [2,3].

The process of adding methyl radicals (CH_3_) to carbon 5 of cytosines is dependent on enzymes from the DNA methyltransferases (DNMTs) family. In the literature, three members of this family have been well documented: DNMT1 is a maintenance methylase, that acts during DNA replication in hemimethylated strands to maintain the methylation profile of the original cell. DNMT3A and DNMT3B are de novo methylases, which add the methyl radical to CpG sites without previous methylation marks [1]. The methyl radical donor for DNMTs is the S-adenosylmethionine (SAM) molecule and it derives from the metabolism of folate, vitamin B12, methionine, and choline [4].

The demethylation process is catalyzed by another family of enzymes, called ten-eleven translocation (TET) with three members: TET1, TET2, and TET3. They catalyze demethylation reactions by oxidation of 5-mC and promote locus-specific reversion of methylation [1,5]. In this process, 5-mC oxidation products are generated (5-hydroxymethylcytosine, 5-hmC; 5-formylcytosine, 5-fC; 5-carboxylcytosine, 5-caC). These products are intermediates in the conversion of 5-mCs into unmethylated cytosines in a process called active demethylation. Demethylation can also be a passive process that is independent of an enzymatic reaction, in which the loss of methyl radicals is due to failures in the maintenance of methylation carried out by DNMT1 [5].

Epigenetic marks change with age, thus gene expression modulates throughout life (DNA methylation clock) [6]. It is known that countless environmental factors and individual habits can alter the DNA methylation profile and, as a result, influence gene expression and possibly contribute to ageing, as well as the development of tumors, inflammatory diseases, or even mental illnesses [7,8,9,10].

Among the environmental factors that potentially alter the DNA methylation profile already described in the literature, ultraviolet (UV) radiation is one of the least explored. Although there is evidence that UV radiation causes photoaging and may cause mutations leading to tumor development (UV Signature Mutations) [11,12], little is known about the ability of UV radiation to cause epimutations, which are changes in the DNA methylation profile, and its association with photoaging and disease (UV Signature epimutations).

### 1.2. Ultraviolet Radiation (UV Radiation) and Epidermal Tissue

UV radiation is emitted by sunlight and lies between the spectrum of electromagnetic radiation from X-rays and visible light, with wavelengths ranging from 100 to 400 nm. UV light is divided into three bands according to wavelength: UVA (320–400 nm), UVB (290–320 nm), and UVC (100–280 nm). Due to the atmospheric ozone layer that blocks UVC and most UVB rays, the radiation reaching the Earth’s surface is a mixture of UVA (90–95%) and UVB (5–10%). UV radiation can also be artificial, that is, be emitted by man-made UV lamps. As synonyms for UV radiation, the terms UV light or UV rays are often used [13].

UVA and UVB radiation have important health implications. Solar radiation is necessary for the synthesis of vitamin D, however, long periods of exposure and for many years (chronic exposure) can lead to edema, erythema, burns, immunosuppression, and skin cancer [14,15]. UV radiation can cause direct biological damage or indirect damage by the production of reactive oxygen species (ROS). UVB is absorbed by DNA and induces two main types of DNA damage: cyclobutane-pyrimidine dimers (CPDs) and (6-4) pyrimidine-pyrimidone photoproducts ((6-4) PPs). UVA, in turn, induces reactive oxygen species that cause oxidative DNA damage (such as 8-oxo- deoxyguanosine) [16,17].

Because sunlight is ubiquitous, all humans are potentially exposed to it, and the skin is the most exposed organ. The skin is composed of the epidermis, dermis, and hypodermis. The epidermis corresponds to the most superficial layer and therefore, the most exposed to environmental factors. The mammalian epidermis is composed of stratified epithelium, representing the protective, outermost barrier of the body. Epidermal cells, commonly called keratinocytes, densely pack the epidermis to a depth between 75 and 150 μm, reaching up to 600 μm in thickness on palms and soles [18].

Five layers (strata) form the epidermis, and they are largely defined by the characteristics of their keratinocytes such as size, shape, nucleation, and expression of keratin: stratum corneum, lucid stratum, granular stratum, spinous stratum, and germinative stratum. These sublayers represent different stages of maturation of the keratinocytes generated from the epidermal stem cells and progenitor cells that actively divide in the basal layer. The epidermal structure changes from anuclear cells in the stratum corneum (surface layer) to distinct hexagonal-shaped cells in the basal layer (deep layer). The basal stratum is made up of keratinocytes that proliferate and push existing cells to an upper layer. The stratum corneum, composed of several laminated and loosely bound keratinized cells, provides an important barrier function protecting the underlying layers. Melanocytes reside in the basal stratum and produce melanin, a pigment which protects the skin from UV radiation. UVA rays reach the deepest layers of the skin and can cause elastosis, while UVB rays predominantly cause erythema or sunburn [14,15,18].

During tissue homeostasis, stem cells and epidermal progenitors achieve a balance between self-renewal and differentiation, as they need to replace keratinocytes in the stratum corneum desquamated by newly differentiated cells migrating from the base of the epidermis [18,19,20].

The epidermis has one of the highest cell turnover rates in the body, which makes it one of the tissues most likely to develop tumors [20]. In fact, the incidence of epidermal cancer (traditionally known as skin cancer) has been increasing, with non-melanoma skin cancer (also known as keratinocyte carcinoma: basal or squamous cell carcinoma) being the most common malignancy worldwide [14,15,17,20]. An increase has been reported in United Kingdom [21], the United States [15], Brazil [22], Australia [23], and China [24]. It is estimated that UV radiation is the cause of approximately 65% of melanomas and 90% of non-melanomas [15].

The essential environmental issue involved in the increasing incidence of skin cancer is the depletion of the stratospheric ozone layer, and it is one of the most critical environmental topics of the past 40 years. By absorbing part of the UV radiation, the ozone layer prevents biological damage such as aberrations in the DNA methylation profile (epimutations), commonly reported in skin cancer [25,26,27]. This indicates that chronic exposure to UV radiation may cause an imbalance in epigenetic homeostasis.

### 1.3. DNA Methylation in Epidermal Homeostasis

Two recent reviews [28,29] reported that DNA methylation, alongside with other epigenetic marks such as chemical modifications in histones, cooperate to modulate the transcription of genes and, consequently, epidermal homeostasis during the differentiation of epidermal cells. DNA methylation is essential for the development of mammals. Embryonic stem cells and adult epidermal stem cells undergo extensive changes in the DNA methylation profile during in vitro differentiation [30,31,32,33].

A study of primary culture of human keratinocytes showed that, during the differentiation process, more than 50% of the genes suffered a loss of methylation (hypomethylation) while other genes underwent hypermethylation [34]. Some examples of genes that have undergone hypomethylation are the *S100P, LCE3D, POU2F3, MAFF*, and *SP1* genes which are all responsible for encoding transcription factors involved in the cell differentiation process [29]. Later on, in a study with different cell types of mouse epidermis, Bock et al. [32] revealed that the differentiation of adult stem cells causes a general reduction in methylation on regulatory elements specific to some lineages, while those related to other lineages became increasingly methylated.

It was also shown that during the differentiation of epidermal cells, there is a relationship between the methylation profile and the expression of DNMTs. While DNMT1 became negatively regulated during the process, DNMT3A and DNMT3B were up- and down-regulated, respectively [33,34]. Overall, these data show that DNA methylation is an important and essential mechanism for epidermal cell differentiation, which suggests that changes in this profile can disrupt epidermal homeostasis, contributing to the development of diseases and photoaging [28,29].

It is known that epidermal homeostasis can be disturbed by pathogens [35] and environmental factors such as UV radiation [14,15,17]. The latter is the focus of the present study, which aims to review the literature concerning the influence of UV radiation on the DNA methylation profile in epidermal cells, as well as on skin samples, in an attempt to synthesize data on the association between these cells’ DNA methylation profile and UV radiation exposure.

## 2. Results and Discussion

The included articles are described in Table 1, Table 2 and Table 3. They are organized by order of publication and comprise studies that focused on the influence of artificial (man-made lighting) or natural (solar) UV radiation, both in cultured and human/animal skin-derived cells.

Most studies in Table 1 show data for HaCat cells (normal human keratinocyte cell line derived from human skin), a widely used model for studies concerning epithelial cells. The majority of these studies focused on the effect of artificial UVA or UVB radiation on the DNA methylation profile. Studies based on site-specific methylation showed an interest in tumor suppressor genes, such as *p16* and *RASSF1*, and their data reveal that UV irradiation was able to increase the level of methylation in the studied genes [36,37,39]. Studies in which expression analysis was performed showed an agreement between the hypermethylated profile and decreased transcript levels of tumor suppressor genes [37,39].

For global methylation analysis, the studies did not detect changes between irradiated and non-irradiated cells [38,40]. One study detected an increase in global hydroxymethylation levels, which was found to align with levels of transcripts and demethylases from the TET family. Another study evaluated the effect of a polypeptide from *Chlamy Farreri* (PCF) upon the methylation profile of irradiated cells. UV radiation was used to induce carcinogenesis, and hypermethylation of tumor suppressor genes was detected. Decreased transcript levels of these genes were reported, and the polypeptide was able to reverse the aberrant methylation profile [39]. 

With regard to other cell lines, a study with HDK1 cells (immortalized human epidermal cell line) reported hypomethylation of *WNT1* (oncogene), which was found to be in agreement with an increase in its level of transcripts in irradiated cells [41].

Studies involving animal models are compiled in Table 2. Most focused on the effect of a compound on the skin of UV-irradiated animals, which was used to induce carcinogenesis [42,43,44,46]. UV radiation-induced carcinogenesis was associated with changes in the DNA methylation profile, either hyper or hypomethylation in global or site-specific scenarios [42,43,45,46]. Large-scale DNA methylation studies revealed changes in the methylation profile of genes involved with the cell cycle and carcinogenesis [43,45,46]. Studies in which expression analysis was performed showed an agreement between the global/site-specific methylation profile and the level of transcripts of some of the studied targets [42,44,46]. Global methylation studies were inconsistent, as one revealed an increase in the levels of 5-mC [44] and another revealed a decrease [42], which was in line with higher or lower levels of DNMT transcripts, respectively. The discrepancy can be explained by methodological differences between the chosen animal model, cell type (normal or tumor), and amount/time of UV irradiation. In all studies, compounds used topically on the animals’ skins were able to inhibit the unwanted profile (when applied alongside with UV irradiation) [42] or revert to the desired DNA methylation profile (when applied after the irradiation by UV light) [44,46].

Table 3 shows data from studies with human skin samples, and in most of these studies, the studied radiation was natural (solar) [47,49,50,51]. In these cases, it is not possible to measure the level of exposure for volunteers precisely, and samples were collected from areas exposed and not exposed to the sun, such as the inner and outer regions of the arm. Nevertheless, these studies revealed changes that occurred naturally throughout life in terms of the amount of sun exposure. Two studies showed global hypomethylation and both hyper and hypomethylation at specific sites in the epidermis; these data were also associated with age (photoaging), meaning that the older the volunteer (which indicates more time of sun exposure), the greater the level of change [47,49]. A study also revealed differences between the profile of the dermis and the epidermis [47]. However, two other studies did not show the influence of radiation on the global or site-specific methylation profile [50,51]. 

The difference between these studies can be explained by the fact that in the first two [47,49], there was a separation of the dermis and epidermis, and in the others [50,51], total skin samples were used. Different cell types can respond differently to environmental factors; therefore, while the tissue was more homogeneous in studies with separation of dermis and epidermis regarding the cell type, possible changes may have been diluted in total skin samples since they contain several different cell types in a heterogeneous mass of cells. Still, in studies where alterations were observed (ethnic differences: Fitzpatrick phototypes), the analyzed skin type was Caucasian, therefore presenting phototype II (clear) [47,49,52]. In contrast, in those where no differences were found, phototypes III, IV, and V (light, moderate, and dark brown, respectively) were evaluated [50,51,52]. 

Moreover, a study analyzed the effect of radiation (phototherapy) in psoriasis treatment and revealed that it was able to change the skin’s initially hypomethylated profile of the psoriasis group to a profile similar to that of the healthy control group [48].

The influence of UV radiation on the DNA methylation profile was not observed in all analyzed studies/genes, showing that some CpG sites may be more susceptible to change than others, or that the level and/or time of exposure was not able to cause such changes. In addition, as discussed earlier, methodological differences may also have contributed to contrasting results. Furthermore, differences in the hypermethylated or hypomethylated profile in distinct regions of the genome (some genes were hypermethylated, while others were hypomethylated in the same study) show that radiation has the ability to change the methylation profile in a particular way in different CpG sites.

While studies with cells in culture and animals can be controlled, studies with humans are more challenging since it is impossible to know the volunteer’s exact level of exposure to radiation. Also, the use of sunscreen and clothing may filter radiation [14,15,17]. In such studies, the participant’s or family’s statement was the only available information. It is also necessary to consider skin type (Fitzpatrick phototypes), which is known to influence a person’s susceptibility to the effects of solar radiation [15]. This may also be true for epigenetic marks, since epigenome-wide studies reported variations in methylation patterns between populations, including Caucasians, non-Caucasians (dark-skinned), Hispanics, Arabs, and Africans [53].

Data on changes in the DNA methylation profile derived from UV radiation suggest that epigenetic changes may contribute to the breakdown of cellular/tissue homeostasis and be an important event in the development of skin diseases, such as cancer. In fact, animal studies show that the induction of carcinogenesis by UV radiation involves epigenetic changes [42,43,45,46]. On the other hand, one study showed that phototherapy can be a strategy for treating psoriasis, suggesting that UV radiation can also be a tool for disease treatment. The photobiological effects thus achieved depend on the wavelengths used. Other studies showed that the DNA methylation profile responded to chemical treatments—such as PCF, Honokiol, triterpenoid ursolic acid, and epigallocatechin-3—indicating that these epigenetic marks are potential targets for skin disease treatment [39,42,44,46].

## 3. Materials and Methods 

### 3.1. Literature Search Strategy

The main search terms used were UV radiation/sun exposure AND skin/epidermis AND DNA methylation, followed by a definition of their respective MeSH terms and synonyms (entry terms). The literature review considered all articles published until 22 June 2020, and indexed in four databases (PubMed, Scopus, Web of Science, and Cochrane). References were compiled in a reference management tool (Mendeley v1.19.6) for title and abstract selection based on inclusion and exclusion criteria.

### 3.2. Inclusion and Exclusion Criteria

To be eligible for inclusion, studies had to evaluate the effect of ultraviolet radiation (UVA, 400–320 nm; UVB, 320–280 nm; and UVC, 280–100 nm) on the DNA methylation profile of epidermal cells derived from cell cultures, animal models, and/or human skin. Interventional and observational studies were included without the restriction of language or date of publication. 

**Figure 1:**
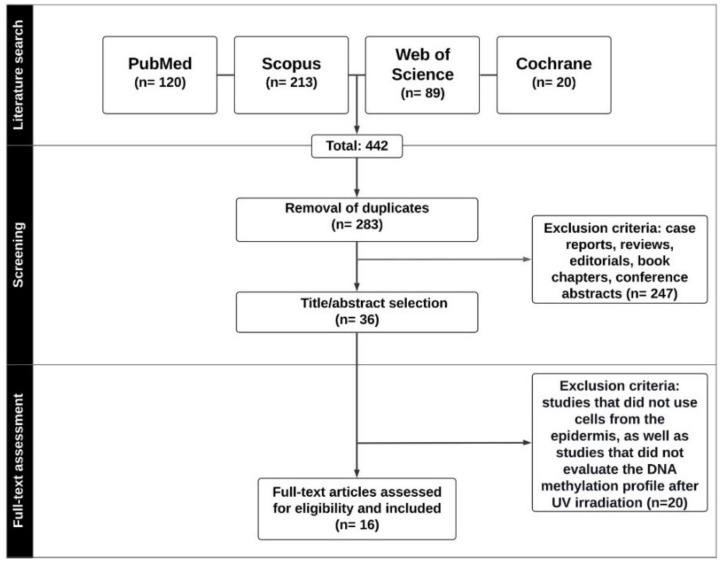
Prisma diagram containing the article selection flowchart.

Any scientific texts other than research articles (Figure 1) or studies with analysis of DNA methylation levels for any purpose other than identifying irradiation-induced alterations, and studies with cell types different from those of the tissue of interest (epidermis) for the present literature review were excluded. 

### 3.3. Data Extraction 

After duplicate removal, a total of 283 studies were identified as a potential for inclusion in this review. Following title and abstract selection by two independent reviewers (B.F.S. and M.C.C.), 36 articles were assessed for full-text reading, and 20 were excluded due to eligibility issues. Finally, 16 studies were classified as relevant for data extraction. The following data were extracted from the selected articles: authors, country, and year of publication; methodological aspects (country and year of publication, study design, cell type and population, UV radiation treatment protocol, type of DNA methylation and technique used for this analysis); outcomes; and conclusion (Figure 1).

## 4. Final Considerations and Future Perspectives

In conclusion, the data reported here, without any intention of exhausting the topic, show that UV radiation is associated with changes in the DNA methylation profile. These marks are potential biomarkers for environmental exposure and skin diseases, therefore acting as possible targets for treatment, due to their reversibility.

DNA methylation has been widely studied in several contexts, and its potential as a biomarker for environmental exposure and disease diagnosis/prognosis is a consensus in the literature [54,55]. Although aberrant methylation has been detected in inflammatory skin diseases, such as psoriasis and atopic dermatitis, the study of epigenetic marks in this context has not been fully explored as shown in a recent review [56]. DNA methylation has been studied in more detail in skin cancer and the characteristic profile has been found (cancer methylome): global hypomethylation and hypermethylation in tumor suppressor gene promoters [29,57,58].

Furthermore, studies that focused on reversing the DNA methylation profile brought to light a class of drugs called epidrugs, which lead to changes in gene expression. Some DNMT inhibitors (DNMTi), such as Azacitidine (Vidaza) and Decitabine, have already been approved by the FDA and are being used in leukemia and myelodysplastic syndrome treatments. Numerous other drugs go beyond DNMTi, such as inhibitors of histone-modifying enzymes, and have either been approved or are being tested in phase I, II, or III of clinical trials [59]. 

Finally, yet importantly, it should be noted that UV radiation is used for treating diseases [60], and research cited herein reported that DNA methylation can be one of the mechanisms involved in the reestablishment of tissue homeostasis in the context of psoriasis [48].

We are still far from understanding the mechanisms involved in the disruption or reestablishment of epigenetic homeostasis derived from UV radiation on epidermal cells or total skin. However, studies conducted so far (2003–2019) suggest that this context is an important area of study that needs to be addressed and further explored in order to understand: (i) how epigenetic marks can predict UV radiation-derived alterations and skin disease/photoaging progression; (ii) which marks can be used for early diagnosis or respond to epidrugs; (iii) how different skin types (Fitzpatrick phototypes) behave when submitted to UV radiation, with respect to epigenetic marks; (iv) which epidrugs can be used on epidermal tissue/skin; and (v) which wavelengths disrupt or reestablish epigenetic homeostasis and how it is done. All of these questions will assist in the development of precision medicine, which provides personalized treatment for the patient [61]. Overall, the data reported here suggest that epigenetic homeostasis can be disrupted or restored by exposure to UV radiation. Hence, it is essential that this context be further explored in an attempt to understand better the association of DNA methylation profile in epidermis/skin and exposure to UV radiation.

## Figures and Tables

**Table 1:** Compilation of studies with cell lines exposed to UV radiation and DNA methylation

Country, Year [Reference]	Study Design	Cell Lines	Groups	UV Radiation Treatment	Methylation Type	Technique	Outcome and Conclusion	Other Outcomes
India, 2006 [36]	Comparison of UVA and UVB-irradiated and non-irradiated cells	HaCaT and A431 (epidermoid carcinoma)	Irradiated group x non-irradiated group	6 cycles of exposure to UVA (150–200 mJ/cm^2^) and UVB (15–20 mJ/cm^2^). Cells were cultured for 1–2 days before re-exposure to the same radiation dosage	Site-specific (*p16, MGMT, DAP Kinase, GSTP1*)	COBRA	Hypermethylation of *p16* was observed on the irradiated group	
Germany, 2011 [37]	Comparison of UVA-irradiated and non-irradiated cells	HaCaT	Irradiated group x non-irradiated group	UVA exposure (200 kJ/m^2^) once a week for 10 to 15 weeks, with a six-day interval between irradiation events	Site-specific (*p16*)	qMSP	Hypermethylation of *p16* (up to 70%) as well as ↓ of its transcripts on the irradiated group	
USA, 2013 [38]	Comparison of cells submitted to different doses of radiation, as well as different periods of UV exposure and cell recovery	NHEK	Negative controls (non-irradiated cells); positive low and high-dose controls (one-time radiation); cells exposed to low or high doses of radiation with an 8 or 18-day growth period	10 cycles of low (130 J/m^2^) or high-dose (260 J/m^2^) UVB irradiation with 2–3 days of “cell recovery” between each cycle. After the final irradiation, cells were grown for 8 or 18 days	Global and site-specific (*CXXC5, PPP3CB, L17C, CCDC40, C21orf29*)	MIRA combined with microarray analysis; COBRA	No differences were detected in the DNA methylation profile of irradiated cells. Further studies are encouraged.	
China, 2015 [39]	Evaluation of PCF effect upon DNA methylation in UVB-irradiated cells	HaCaT	Non-irradiated group (control); irradiated group, no treatment x irradiated group + PCF or vitamin C	20 chronic UVB exposure cycles (10 mJ/cm^2^ for 15 min per cycle) with 24–48h intervals	Site-specific (*p16, RASSF1A*)	MS-HRM	Hypermethylation of *p16* and *RASSF1A*, ↓ transcript levels of both genes, and ↑ in transcript and protein levels of DNMT3B in irradiated cells	PCF provoked demethylation on the studied tumor suppressor genes and generated better effects than vitamin C
China, 2017 [40]	Comparison of UVB-irradiated and non-irradiated cells	HaCaT	Irradiated x non-irradiated cells	UVB exposure (40 mJ/cm^2^ or 80 mJ/cm^2^) for 24h	Global methylation and hydroxymethylation	IHC; IF	↑ Global hydroxymethylation and ↑ transcript and protein levels of TETs 1, 2 and 3 in irradiated cells	
Japan, 2019 [41]	Comparison of UVB irradiated and non-irradiated cells, as well as between UV-exposed and non-exposed human facial regions	HDK1 and cells from human facial biopsies	Exposed group x non-exposed group	Exposure to different doses of UVB (10 or 100mJ/cm^2^) for two weeks (4× or less per week) for HDK1; regular sun exposure for facial samples	Global and site-specific *(WNT1)*	IHC; BS	Hypomethylation of *WNT1* dose-dependent and ↑ transcript levels of *WNT1* in irradiated cells.	Hypomethylation of *WTN1*; ↓ global methylation; ↓ DNMT1 levels in solar lentigines

COBRA: Combined bisulfite restriction analysis; qMSP: Quantitative methylation-specific PCR; MIRA: Methylated-CpG island recovery assay; MS-HRM: Methylation specific-high resolution melting analyses; HC: Immunohistochemistry; IF Immunofluorescence; BS: Bisulphite sequencing.

**Table 2:** Compilation of studies with animal models exposed to UV radiation and DNA methylation

Country, Year [Reference]	Study Design	Animal Model and Groups	Tissue or Cell Type	UV Radiation Treatment	Methylation Type	Technique	Outcome and Conclusion	Other Outcomes
USA, 2003 [42]	Effect of Epigallocatechin-3-gallate (EGCG) on the skin of mice submitted to a UV-induced carcinogenesis protocol	SKH-1 mice divided into 3 groups: non-irradiated (control), irradiated and irradiated + EGCG	Cells from dorsal skin biopsies (papillomas and carcinomas) and epidermal cells from the non-irradiated group	UVB (180 mJ/cm^2^); irradiation once a day for 10 days (tumor initiation); then 3× a week for 30 weeks (tumor promotion)	Global	IHC	Global hypomethylation and ↓ in DNMT1 activity on both irradiated groups	ECGC prevented global hypomethylation in the treated group
USA/South Coreia, 2014 [43]	Comparison of methylation profiles from skin tumors submitted to irradiation and DMBA/TPA-induced carcinogenesis	SKH-1 female mice from 7 to 8 weeks of age irradiated and non-irradiated (control) with UVB; CD-1 female mice of 6 weeks of age treated with DMBA/TPA	Cells from biopsies of dorsal skin tumors (papillomas and carcinomas); epidermal cells from the control group	UVB (280–320 nm; 70–80% of total energy); UVA (320–375 nm; 20–75% of the total energy). Mice were exposed to UV light (30 mJ/cm^2^) twice a week for 36 weeks	Whole-genome	MeDIP-Seq; IPA	Hypermethylation in 4140 genes and hypomethylation in 1863 from the UV group (sequences involved in the molecular mechanism of cancer were the most affected)	DMBA/TPA promoted an altered methylation profile when compared to controls.
USA, 2017 [44]	Effect of Honokiol (HK) on the skin’ methylation profile of both irradiated and non-irradiated	C3H/HeN female mice (5 to 6 weeks); 129 Ola/C57BL/6 mice, both COX-2 deficient and wild type. Groups were divided in non-irradiated (control), irradiated and irradiated + HK in two different concentrations (4% and 8%)	Skin biopsies from shaved dorsal region	Mice were irradiated with 150 mJ/cm^2^ of UVB radiation (280–320 nm; ≈ 80% of total energy) for 4 consecutive days and sacrificed 24 h after the last irradiation session	Global	IF; ELISA; Dot-blot analysis	Higher numbers of 5mC-positive cells; ↑ expression of Dnmt1, 3a and 3b, as well as Sp1 and 3; ↓ TET activity; ↑ global methylation levels in irradiated animals when compared to controls	Topical application of HK inhibited the UVB-induced formation of 5-mC-positive cells in a dose-dependent manner
USA, 2019 [45]	Analysis of methylation profile during different stages of radiation-induced carcinogenesis	SKH1 mice divided into two groups: non-irradiated (control) and submitted to UVB	Epidermal and tumor biopsies from different time points (2, 15 and 25 weeks); whole skin samples from the control group	UVB (60mJ/cm^2^), twice a week for 25 weeks.	Genome-wide, base resolution	MethylSeq; Pyrosequencing	Changes in 974 DMRs: 50% hypomethylated and 50% hypermethylated; methylation and expression are related in 60% of the DMRs (ex: *Cdk4, Tgfbr2, Fgfr1, Bcl2l1, Pik3cb*) in the epidermis of irradiated animals.	
USA, 2019 [46]	Analysis of effect of tripterpenoid ursolic acid (UA) upon the methylation profile of mice submitted to radiation-induced carcinogenesis	SKH-1 hairless mice divided into 3 groups: non-irradiated + acetone (control), irradiated + acetone and irradiated + UA	Epidermal cells from skin samples (control) and skin tumor cells from different time points (2, 15, and 25 weeks)	UVB (60mJ/cm^2^), twice a week for 25 weeks	Genome-wide, base resolution	MethylSeq; Pyrosequencing	Hypermethylation (*Slco5a1, Ogfrl1, Bend6, Mgat4a, Creg2, Gm973, Slc4a3, Arl4c, Mlph, Twist2 Slco5a1, Ogfrl1, Bend6, Mgat4a, Creg2, Gm973, Slc4a3, Arl4c, Mlph, Twist2*) and hypomethylation (*Npbwr1, Plekhb2,Klf7, Mgat5, Ube2t, Phlda3, Kcnj9, Fbxo5, Plagl1,Myb)*; altered transcript levels in agreement with methylation analysis in irradiated animals;	Ursolic acid was able to reverse all observed methylation changes.

IHC: Immunohistochemistry; MeDIP-Seq: Methylated DNA immunoprecipitation sequencing; IPA: Ingenuity^®^ pathway analysis—analysis of function and pathway of methylated genes; IF: Immunofluorescence; ELISA: Enzyme-linked immunosorbent assay; MethylSeq: Methylation sequencing.

**Table 3:** Compilation of studies with human skin exposed to UV radiation and DNA methylation

Country, Year [Reference]	Study Design	Groups	Tissue/Cell Lines	UV Radiation Exposition	Methylation Type	Technique	Outcome and Conclusion	Other Outcomes
Germany, 2010 [47]	Comparison of sun-exposed and non-exposed body areas of healthy individuals	30 volunteers (10 suction blisters for male individuals; 20 punch biopsies for female individuals)	Dermal and epidermal cells from outer forearm and inner arm regions	Lifelong solar radiation	Genome-wide and site-specific	Human Methylation 27 BeadChip; BS (*KRT75, SEC31L2, DDAH2, TET2*)	Hypomethylation in chronically-exposed epidermis; hypomethylation of *KRT5*; ↑ expression levels of *KRT5*; hypermethylation of *SEC31L2*	≠ methylation profiles in male and female skin; age-related hypermethylation in 104 markers; hypermethylation of *DDAH2* and *TET2*; ≠ methylation profile between dermis and epidermis
Sweden, 2015 [48]	Effect of phototherapy upon the skin methylation profile of patients with psoriasis	24 individuals divided into 2 groups: psoriasis patients (n = 12) and healthy subjects (n = 12, control group)	Epidermis cells obtained from punch biopsies	Whole body narrow band (311–312 nm) UVB light irradiation in a cabinet equipped with fluorescent lamps (UVB TL100W/01, Philips); 24 sessions in for 2–3 months	Genome-scale	Human Methylation 450	Hypomethylation on psoriasis group; after phototherapy, the methylation profile was similar to that of the healthy group.	Altering in the methylation profile was associated with a better response to treatment.
USA, 2015 [49]	Comparison of sun-exposed and non-exposed areas of the body in healthy and skin cancer patients	36 individuals <35 and >60 years old divided into 2 groups: healthy subjects (n = 26) and squamous cell carcinoma (SCC) patients (n = 10)	Dermis and epidermis cells obtained by punch biopsies of the arm (upper inner arm) and face (outer forearm or lateral epicanthus)	Lifelong solar radiation	Genome-scale	Human Methylation 450; WGBS	Hypomethylation blocks throughout the genome in sun-exposed regions, mainly in older individuals.	Hypomethylation in sun-exposed regions of healthy volunteers was similar to the profile of individuals with SCC.
Brazil, 2015 [50]	Comparison of sun-exposed and non-exposed areas of the body	Corpses of both genders, over 30 years old and with healthy skin (n = 28)	Skin punch biopsies (dermis + epidermis) from the inner arm or outer forearm	Lifelong solar radiation	Site-specific	MSP (*MMP9, miR-137, KRT14*); MSRE (*KRT19*)	No difference was detected between exposed and non-exposed regions	Methylated profile of *miR-137* seemingly more frequent in women
Brazil, 2017 [51]	Comparison of sun-exposed and non-exposed areas of the body	Corpses from both genders, ranging from 18–89 years old and with healthy skin (n = 24)	Skin punch biopsies (dermis + epidermis) from the inner arm or outer forearm	Lifelong solar radiation	Global and site-specific.	ELISA; MSP (*miR-9-1, miR-9-3, MTHFR*)	No difference was detected between exposed and non-exposed regions	global methylation levels > global hydroxymethylation levels

BS: Bisulfite sequencing; WBSG: Whole-genome bisulfite sequencing; MSP: Methylation specific PCR; MSRE: Methylation sensitive restriction enzyme; ELISA: Enzyme-linked immunosorbent assay.
